# Demographical and Clinical Characteristics, Risk Factors, and Prognosis of Adult Patients with Herpes Zoster in Türkiye: A Retrospective, Multi-Center Study (VARICOMP-Adult Study)

**DOI:** 10.3390/idr17030068

**Published:** 2025-06-11

**Authors:** Esin Senol, Alpay Azap, Selda Sayin Kutlu, Murat Kutlu, Ayse Erbay, Pelin Kocyigit, Emine Colgecen, Ozlem Ozbagcivan, Nilsel Ilter, Funda Yetkin, Serpil Sener, Birsen Mutlu, Rebiay Kiran, Nese Saltoglu, Burhan Engin, Ener Cagri Dinleyici

**Affiliations:** 1 Department of Infectious Diseases, Faculty of Medicine, Gazi University, Ankara 06500, Türkiye; 2 Department of Infectious Diseases, Faculty of Medicine, Ankara University, Ankara 06230, Türkiye; 3 Department of Infectious Diseases, Faculty of Medicine, Pamukkale University, Denizli 20070, Türkiye; 4 Department of Infectious Diseases, Faculty of Medicine, Bozok University, Yozgat 66900, Türkiye; 5 Department of Dermatology, Faculty of Medicine, Ankara University, Ankara 06230, Türkiye; 6 Department of Dermatology, Faculty of Medicine, Bozok University, Yozgat 66900, Türkiye; 7 Department of Dermatology, Faculty of Medicine, Dokuz Eylul University, Izmir 35340, Türkiye; 8 Department of Dermatology, Faculty of Medicine, Gazi University, Ankara 06500, Türkiye; 9 Department of Infectious Diseases, Faculty of Medicine, Inonu University, Malatya 44280, Türkiye; 10 Department of Dermatology, Faculty of Medicine, Inonu University, Malatya 44280, Türkiye; 11 Department of Infectious Diseases, Faculty of Medicine, Kocaeli University, Kocaeli 41380, Türkiye; 12 Department of Dermatology, Faculty of Medicine, Kocaeli University, Kocaeli 41380, Türkiye; 13 Department of Infectious Diseases, Faculty of Medicine, Istanbul University Cerrahpasa, Istanbul 34098, Türkiye; 14 Department of Dermatology, Faculty of Medicine, Istanbul University Cerrahpasa, Istanbul 34098, Türkiye; 15 Department of Pediatrics, Faculty of Medicine, Eskisehir Osmangazi University, Eskisehir 26040, Türkiye

**Keywords:** herpes zoster, shingles, adults, postherpetic neuralgia

## Abstract

**Introduction:** Over the past few decades, the rising incidence of herpes zoster (HZ) rates appears to have been a global phenomenon. In Türkiye, there is a lack of comprehensive studies addressing the HZ burden of disease, risk factors, and clinical characteristics. The aim of the VARICOMP-Adult study was to evaluate the clinical and demographic findings of adult patients with HZ. **Patients and Methods:** We enrolled the medical records of 1955 patients, 1010 females and 945 males, aged between 18 and 97 years between 2009 and 2014. **Results:** The presence of underlying conditions was present in 35.5% of patients and 345 patients (17.6%) had immunosuppression; 18.0% of patients required hospitalization. The mean age, the presence of underlying conditions, and immunosuppression in hospitalized cases with HZ were higher than those in outpatients. Logistic regression analysis revealed the following risk factors for hospitalization: age, immunosuppression, hypertension, hematological disorders, transplantation, COPD, and the presence of HZ opthalmicus or disseminated disease. We observed seven HZ cases with mortality aged between 58 and 80 years, and all cases had an underlying condition; 9.9% of the entire population reported postherpetic neuralgia (PHN), and age and no previous antiviral medications were the risk factors for PHN. **Conclusions:** This is the largest nationwide study of adult patients with HZ. Effective healthcare interventions such as antiviral therapy and immunization could prove beneficial in combating disease and treating HZ complications, especially in the high-risk population and individuals of older ages.

## 1. Introduction

Initial infection with the varicella zoster virus (VZV) typically results in the development of chickenpox, which is most commonly observed in children [1]. Herpes zoster (HZ), commonly known as shingles, occurs when the VZV virus becomes active again after a period of dormancy following the initial infection. Typically, HZ manifests as a painful, localized rash on the skin [2,3]. The general population has a lifetime risk of developing HZ that varies within the range of 20% to 30% [3]. Estimates indicate that the incidence rate of HZ across North America, Europe, and Asia-Pacific ranges between 3 and 5 cases per 1000 person/years [4]. The precise immunological mechanism that regulates the latency of VZV remains poorly comprehended [2]. The likelihood of developing HZ significantly rises with age, greater in adults over the age of 50, particularly among older individuals or those with weakened immune systems (such as individuals with cancer, those who have undergone bone marrow or solid organ transplantation, and those using immunosuppressive drugs) because of the waning in cell-mediated immunity [1,2,3,4,5,6]. Additional risk factors include the female gender, ethnicity, a family history, physical trauma, previous occurrences of HZ, and co-existing medical conditions such as asthma, diabetes mellitus (DM), or chronic obstructive pulmonary disorders (COPDs) [7]. For those who are previously healthy, there are limited systemic symptoms. HZ can spread in individuals with weakened immune systems, leading to widespread skin lesions, and might affect the central nervous system, lungs, and liver [2]. The elderly population commonly experiences a severe course of illness with a high risk of sequelae, particularly the debilitating and long-lasting condition known as postherpetic neuralgia (PHN), as well as the need for hospitalization [4]. Additional short- and long-term problems associated with HZ include secondary bacterial infection, dermatologic complications, acute central nervous system involvement, persistent neurologic consequences, and VZV vasculopathy [3,4,8]. HZ can significantly harm the physical and mental health of patients, particularly older adults. This can lead to poor consequences for their ability to function, maintain independence, and quality of life [3,6].

The increasing frequency of HZ rates appears to have been a global phenomenon over the past few decades, unaffected by changes in demographics such as an aging population and a higher number of immunocompromised individuals [3,5]. Experts anticipate that HZ’s global impact on health will continue to rise in the future [4]. Healthcare practitioners and health officials must rely on the most reliable and current research regarding the burden of disease, treatment, and preventative efforts for HZ [4]. Nevertheless, there is a dearth of extensive research investigating the burden of disease, risk factors, and clinical aspects of HZ in Türkiye on a nationwide scale [9]. The objective of this multicenter study was to assess the demographic and clinical characteristics of adult patients with HZ in Türkiye.

## 2. Materials and Methods

The VARICOMP-Adult study is a multicenter retrospective study conducted in Türkiye to collect epidemiological and economic data on adult patients who were over 18 years old and had HZ infection. The analysis covers the period from 1 January 2009 to 31 December 2014. The study was approved by the Local Ethical Committee of the Ankara University Faculty of Medicine on 28 November 2016 (18-918-16). The methods used in this study complied with the ethical guidelines established by the institutional and/or national research committee, as well as the 1964 Helsinki Declaration and its subsequent amendments or comparable ethical standards. Following authorization from the local ethical committee, we collected medical records of patients diagnosed with HZ from eight centers in seven different cities. Patient confidentiality was strictly maintained throughout the study by anonymizing all personal identifiers, and only de-identified data were used in the analysis. Access to patient data was restricted to authorized study personnel.

In Türkiye, the population was 72,561,312 in 2009 and 77,695,904 in 2014. In this time, 32.7% of the entire population was 18 or below 18 years old, and 11.7% of the entire population was 60 or above 60 years old. There is no mandatory notification of HZ, and there is no official registration system. Each study center thoroughly evaluated their medical records to identify patients over 18 years old throughout the entire trial period. In hospitals or outpatient settings, we used International Statistical Classification of Diseases and Health-Related Problems (ICD-10) diagnostic codes for HZ. The study included both adult patients who were previously healthy and those with pre-existing underlying illnesses.

We documented the admission time, including the year and month, age, sex, and any pre-existing underlying problems. We also collected data on the involvement of the dermatome, the presence and length of hospitalization (in days, if known), the necessity for admission to an intensive care unit and duration of stay (in days, if known), specifics of antiviral, local, and/or systemic antibiotic treatment, and the outcome of hospitalization and mortality. We also collected data on conditions such as hypertension, diabetes mellitus, hematological disorders (including hematological malignancy, excluding bone marrow transplantation), solid tumors, and transplantation (including bone marrow or organs). We enrolled HZ patients who had been diagnosed during pregnancy. We evaluated patients with multiple diseases separately in their respective categories.

We assessed each patient’s immune status during the HZ episode by recording the presence of diseases and medications associated with “immunosuppression”, such as actively treated malignancies, all hematological malignancies, transplantation, HIV, and any other form of immunosuppressive therapy [5,10]. Postherpetic neuralgia (PHN) is a condition characterized by persistent pain in the region previously affected by HZ, persisting for more than 90 days after the rash appearance [11]. The condition known as herpes zoster ophthalmicus (HZO) refers to HZ that specifically affects the ophthalmic division of the trigeminal nerve [11].

The primary aim of this study was to determine the age distribution and presence of underling conditions, dermatome involvement, and prognosis of HZ-affected individuals. The secondary endpoint is to define risk factors related to hospitalization and PHN rates. We also plan to evaluate the clinical and demographic characteristics of patients who died during their follow-up.

We utilized the JASP statistical analysis application (JASP 0.16.4 version, JASP Team, Amsterdam, the Netherlands) to perform the statistical analyses. We presented the qualitative variables as frequencies and represented the quantitative variables by their mean value plus or minus the standard deviation if normally distributed, or by the median (interquartile range) if not normally distributed. We used independent t-tests to compare continuous data that followed a normal distribution and employed Mann–Whitney U tests to evaluate data that did not follow a normal distribution. We assessed the relationships between qualitative variables using a chi-square test. We computed odds ratios (OR), with the outpatient group, patients without complicated PHN, or patients without immunosuppression serving as the reference group. We performed stepwise logistic regression using backward elimination to identify independent risk factors associated with hospital-acquired HZ, immunosuppression, and PHN among hospitalized patients. The candidate variables for multivariable analysis were selected based on both statistical and clinical criteria. Variables deemed clinically relevant were included in the initial multivariable model. Goodness-of-fit was evaluated using the Hosmer–Lemeshow test.

We considered a *p*-value less than 0.05 as statistically significant.

## 3. Results

The study included 1955 HZ patients (1010 females and 945 males) who were followed up between 2009 and 2014. The mean age was 55.0 ± 17.9 years and ranged between 18 and 97 years. In our study, there was no seasonal variability during the 5-year study period. Table 1 summarizes the gender distribution, age groups, presence of underlying disease, and immunosuppression, both overall and for each study year. The presence of an underlying condition was present in 695 (35.5%) patients, and the presence of immunosuppression was present in 345 patients (17.6%). The distribution of underlying diseases was as follows: 6.1% hematological disorders (n = 120), 6% solid tumors (n = 118), 5.2% diabetes mellitus (n = 101), 7.5% hypertension (n = 146), 1.9% transplantation (organ or bone marrow; n = 37), 2.7% coronary artery disease (n = 53), 2.3% chronic obstructive pulmonary disease (n = 46), 2.9% rheumatological diseases (n = 58), 1.2% endocrinological disorders (except diabetes mellitus; n = 23), 0.6% stroke (n = 12), 0.3% inflammatory bowel disease (n = 6), 0.5% liver disorders (n = 10), 1.3% kidney disorders including chronic renal failure (n = 26), and 1.12% neurological disorders (n = 22). There were four pregnant women and one patient with HIV. The age distribution and percentage of underlying conditions are similar between each study year (Table 1).

Thoracal dermatomes were mostly involved (50.3%), followed by cervical (19.4%), lumbar (11.7%), and sacral dermatomes (5.6%). Trigeminal involvement has been observed in 42 patients (2.1%) and HZO in 73 patients (3.7%); 16 cases have been noted as disseminated disease (Table 1).

In total, 353 patients were followed up in the hospital and 10 patients (0.51%) required an intensive care unit stay; 331 patients (16.9%) received acyclovir, 836 patients received valacyclovir (42.7%), 313 patients received brivudine (16.0%), and 455 patients did not receive antiviral medications; 927 patients (47.4%) received local antibiotic treatment for zona lesions and 98 patients (50%) received systemic antibiotic treatment. The median length of hospital stay was 8 days. Seven patients (0.35%) died despite all interventions.

Underlying diseases, dermatome involvement, treatment, and prognosis of HZ patients, categorized by age groups, have been summarized in Table 2. The age distribution of the patients according to age groups was as follows: 252 patients in the 18–29 year group (12.9%), 182 patients in the 30–39 year group (9.3%), 211 patients in the 40–49 year group (10.8%), 397 patients in the 50–59 year group (20.3%), 475 patients in the 60–69 year group (24.3%), 293 patients in the 70–79 year group (15.0%), 134 patients in the 80–89 year group (6.9%), and 11 patients in the >90 year group (0.6%). Hypertension, diabetes mellitus, coronary artery disease, and chronic obstructive pulmonary disorders have been relatively common in patients over 50 years old, as expected. There are no differences in dermatome involvement between age groups. Immunosuppression was also common in patients in the 40–79 age groups, compared to the 18–29 age group and the 30–39 age group. Hospitalization was also common in age groups over 50 years old compared to those under 50 years old. No patients required an intensive care unit stay below 50 years old and no mortality was observed in this age group. Antiviral use, as well as local and systemic antibiotic use, were similar between the age groups.

### 3.1. Immunosuppression

As shown in Table 3, 345 patients (17.6%) exhibited immunosuppression. The mean age of HZ patients with immunosuppression was higher than that of HZ patients without immunosuppression (57.9 ± 14.6 years vs. 54.3 ± 18.5 years, *p* < 0.001). Men outnumbered 1.15 in HZ patients with immunosuppression, while females outnumbered 1.12 in the outpatient group (*p* < 0.05). The presence of immunosuppression was higher in the 50–59-year-old group, the 60–69-year-old group, and the 80–90-year-old group compared to patients without immunosuppression in the same age groups (*p* < 0.01, *p* < 0.01, and *p* < 0.05, respectively). In terms of immunosuppression status, there is no difference in dermatome involvement. Disseminated disease courses have been commonly seen in patients with immunosuppression. Hospitalization and acyclovir use are also common in patients with immunosuppression (*p* < 0.001 for both) (Table 3).

### 3.2. Hospitalized Patients

Table 4 summarizes comparisons between hospitalized patients (n = 355) and outpatients (n = 1600) based on age, gender, underlying disorders, dermatome involvement, treatment, and prognosis. The mean age of hospitalized patients was higher than that of outpatients (59.9 ± 17.4 years vs. 53.9 ± 17.9 years, *p* < 0.001). Men were outnumbered by 1.2 in the hospitalized patient group, while females were outnumbered by 1.13 in the outpatient group (*p* < 0.01). Immunosuppression and the presence of an underlying condition were significantly common in hospitalized patients (*p* < 0.001 for both). Hypertension, diabetes mellitus, hematological disorders, solid tumors, transplantation, coronary artery disorders, COPD, rheumatological disorders, and renal and neurological disorders were also common in hospitalized patients. There is no difference in dermatome involvement between hospitalized and outpatient groups except for ocular involvement (9.6% vs. 2.4%) and disseminated cases (3.9% vs. 0.12%). Both are common in hospitalized patients (*p* < 0.001 for both). There is no difference in PHN between hospitalized and outpatient cases (8.7% vs. 10.2%; *p* > 0.05). Acyclovir use was common in hospitalized patients (52.3% vs. 9.0%; *p* < 0.001), while valacyclovir (46.4% vs. 26.2; *p* < 0.001) and brivudine (18.5% vs. 1.9%; *p* < 0.001) were common in outpatient cases. Topical and systemic antibiotic use are also common in hospitalized patients (*p* < 0.001 for both) (Table 4). We conducted a logistic regression analysis using the backward LR method, incorporating variables such as age, gender, immunosuppression, hypertension, diabetes mellitus, hematological disorders, solid tumors, transplantation, coronary artery disease, COPD, rheumatological disorders, disseminated disease, and HZO, to identify the factors that influence hospitalizations associated with HZ. The risk factors affecting hospitalization were age (OR 1.011; 1.002–1.009, *p* < 0.05), presence of immunosuppression (OR 4.894; 3.465–6.912, *p* < 0.001), having hypertension (OR 3.428; 2.222–5.287, *p* < 0.001), having hematological disorders (OR 1.794; 1.117–2.883, *p* < 0.05), transplantation (OR 2.748; 1.313–5.752, *p* < 0.01), having COPD (OR 6.514; 3.436–12.351, *p* < 0.001), presence of HZO (OR 5.082; 3.018–8.558, *p* < 0.001), and presence of disseminated disease (OR 14.918; 3.091–71.986, *p* < 0.001).

### 3.3. Postherpetic Neuralgia (PHN)

There were 195 patients with postherpetic neuralgia. Table 5 summarizes comparisons between patients with PHN or without PHN based on age, gender, underlying disorders, dermatome involvement, treatment, and prognosis. The mean age of patients complicated with PHN was higher than patients without PHN (63.5 ± 13.8 years vs. 54.0 ± 18.1 years, *p* < 0.001). There is no difference in gender distribution, the presence of immunosuppression, or underlying disorders. Dermatome involvement, hospitalization rate, and local and systemic antibiotic use were also similar between the groups. Previous acyclovir use (17.6% vs. 10.7%) and brivudine use (16.8% vs. 8.2%) were more common in patients without PHN than in patients with PHN (*p*< 0.05 and *p* < 0.01, respectively). No antiviral use is common in patients with PHN (38.9% vs. 21.5%; *p* < 0.001) (Table 5). Logistic regression analysis was performed with the variables of age, gender, presence of immunosuppression, hypertension, diabetes mellitus, hematological disorders, solid tumors, transplantation, coronary artery disease, COPD, rheumatological disorders, disseminated disease, and HZO to determine the factors affecting PHN. Logistic regression analysis showed that age (OR 1.037; 1.027–1.047, *p* < 0.001) and no previous antiviral medications (OR 2.363; 1.721–3.235, *p* < 0.001) were the risk factors for PHN.

### 3.4. Clinical Characteristics Associated with Patient Mortality

From 2009 to 2014, a total of seven individuals (0.36% of the entire study population)—four male and three female—who were between the ages of 58 and 80 experienced mortality. The initial case was an 80-year-old man suffering from COPD and coronary artery disease. The patient had an HZ infection affecting the thoracic dermatome. The patient died after being hospitalized for 15 days. The second case was a 66-year-old man who had lung cancer, diabetes mellitus, and coronary artery disease. HZ affected his thoracic dermatome, leading to his death after a 63-day hospitalization. The third case was a 62-year-old woman who had diffuse large cell lymphoma and disseminated HZ. She died after being hospitalized for 58 days. The fourth case was a 65-year-old man with colon cancer, lung metastases, and HZ, which affected the lumbar dermatome, who died after 56 days of being hospitalized. The fifth patient, a 58-year-old man with multiple myeloma, died after 32 days of hospitalization due to an HZ affecting the cervical dermatome. The sixth case was a 70-year-old man who had decompensated heart failure and an HZ with ocular involvement (specifically, V1 involvement). After being hospitalized for five days, he died. The seventh-to-last case was an 80-year-old woman with a solid tumor and hypertension. She developed HZ affecting the thoracic dermatome and died after a 24-day hospitalization.

## 4. Discussion

Previously, there was a lack of comprehensive national data about HZ in Türkiye. This study, which involved 1955 adult patients with HZ who were either hospitalized or received outpatient care, is the largest in Türkiye. In recent decades, the prevalence of HZ has increased [3]. A comprehensive analysis, comprising 130 studies conducted in 26 countries across North America, Europe, and the Asia-Pacific region, revealed that the incidence rate of HZ ranged from 3 to 5 cases per 1000 person/years [4]. Data collected from hospitalized patients with HZ provided the most accurate estimate [7]. The prevalence of HZ in the general population of Latin American countries, based on a model, varies from 1.95 cases per 1000 people (for individuals aged 15 to 39 years) to 6.18 cases per 1000 people (for individuals aged 55 to 89 years). The incidence of HZ notably rises after the age of 50, reaching a rate of 6–8 cases per 1000 person/years at 60 years of age and 8–12 cases per 1000 person/years at 80 years of age [7]. There is currently no comprehensive data available on HZ occurrence in Turkey [9]. Researchers found a single comprehensive retrospective study that provided HZ incidence rates derived from data collected at two centers in Istanbul. The incidence of HZ among individuals over the age of 17 increased dramatically from 182 cases per 100,000 people in 2011 to 285 cases per 100,000 people in 2019 [12].

HZ can occur at any age; however, the frequency of cases increases as individuals become older [1]. The study found that the average age of patients with HZ was 55.0 ± 17.9 years, with a range of 18 to 97 years. The mean and age distribution exhibited similarity throughout each study year. The HZ incidence rates were consistent across countries, showing a significant increase beyond the age of 50 and continuing to climb in each subsequent decade [4,5]. Kawai et al. [5] assessed a total of 8017 individuals diagnosed with HZ. The average age of the patients was 51.8 ± 23.7 years, with a range spanning from 0.3 to 101.7 years. In comparison to a reference group consisting of individuals with HZ who are under 50 years old, the probability of developing HZ rose by a factor of 2.7 in the 50–59 age group, 4.12 in the 60–69 age group, 5.68 in the 70–79 age group, and 6.64 in patients over 80 years old. In our study, out of all the patients, 65.2% are aged 50 years or older. The largest proportion of cases, 21.1%, are within the age range of 50 to 59 years, while 23.0% are between 60 and 69 years old. Around half of those who reach the age of 85 will have encountered zoster [1]. Our study included 11 individuals who were over the age of 90, which is a significant proportion of the population in this age group. Women had a considerably higher risk of HZ compared to men [5]. Kawai et al. [5] demonstrated that out of 8017 patients with HZ, 58.7% were females, resulting in a female-to-male ratio of 1:1. Another study conducted in the United States between 1993 and 2016 reported a notable increase in the occurrence of HZ in females [13]. In our own study, the number of females exceeded that of males by a factor of 1.07. However, the group of hospitalized patients had a male-to-female ratio of 1:2.

HZ exhibits no seasonal fluctuations and can manifest at any time of the year [2]. When Kawai et al. investigated the relationship between the month and the risk of HZ, taking into account factors such as sex, age, and year, they found no indication of seasonality in HZ [5]. Research has found that HZ infection in Türkiye follows a seasonal pattern, showing the highest proportion in the summer months and the lowest in the autumn [9]. However, in our study, there was no seasonal variability throughout the 5-year study period.

Immunosuppression and other chronic underlying diseases are additional factors that increase the risk of HZ. Individuals with preexisting comorbidities experience more severe disease symptoms and are at a greater risk of developing complications [7]. The prevalence of PHN and subsequent bacterial infections was greater in immunocompromised patients. Our analysis identified immunosuppression in 345 individuals (17.6%). In this group, the average age of patients was higher, the proportion of men was higher, and hospitalization was more common. In our analysis, 35.5% of patients had underlying conditions. Specifically, 7.5% had hypertension, 6.1% had hematological disorders, 6% had solid tumors, 5.2% had DM, 2.9% had rheumatological diseases, 2.7% had coronary artery disease, and 2.3% had COPD. The age distribution and prevalence of underlying diseases are comparable across each study year. Patients above the age of 50 commonly experience hypertension, DM, coronary artery disease, and COPD, as anticipated. In HZ case reports, DM was one of the predominant coexisting medical conditions, often accompanied by hypertension. A systematic literature review and meta-analysis of studies on the risk of HZ in individuals with DM reported that these individuals have a higher risk of developing HZ, which further increases with age [9].

Thoracal dermatomes are the most affected parts in our study, accounting for 50.3% of cases, as shown in previous investigations. Typically, HZ manifests as a rash on one side of the body that follows a single dermatome and does not extend to the opposite side. The sensory ganglia’s reactivation determines whether dermatome involvement is limited to a single dermatome or extends to adjacent dermatomes [3]. In our investigation, there were 16 cases of disseminated HZ (0.8%). There is no variation in the prevalence of disseminated HZ among different study years or age groups. In this study, 87.5% of disseminated cases required hospitalization; disseminated HZ increased hospitalization by 32-fold. Disseminated HZ is more prevalent among patients with weakened immune systems. However, there have also been documented instances of visceral VZV infection in individuals with normal immune function [3]. Patients with disseminated HZ in Brazil ranged in prevalence from 0 to 16% in the high-risk group and from 0.5% to 7.6% in the average-risk population [7]. Our investigation found that 75% of disseminated HZ cases exhibited immunosuppression, which is consistent with earlier findings.

Herpes zoster ophthalmicus (HZO) occurs when HZV reactivation affects the distribution of the trigeminal nerve’s ophthalmic division [4,11]. While the availability of population-based studies is limited, the reported risks of HZO among patients with HZ were found to be between 10% and 15% [4,14]. The prevalence of HZO was found to be 3.7% among all patients in this study. One of the most often mentioned complications in the case studies from Türkiye was HZO [9]. Although the risk of HZO is comparable among different age groups, we observed a greater incidence of cases among individuals aged 50 and above. A comprehensive study conducted in Brazil showed a prevalence of Ramsay–Hunt syndrome among patients of 1.75% [7]. We documented four cases with Ramsay–Hunt syndrome. The occurrence of HZ-related complications, such as HZO and Ramsay–Hunt syndrome, was more frequent in individuals with weakened immune systems compared to those with normal immune function [7].

The hospitalization rates caused by HZ varied between 3% and 35.7% [6]. According to another report, around 1–4% of individuals who contract HZ require hospitalization due to complications [11]. Among the 28 studies that provided data on hospitalization due to HZ, the rates of HZ-related hospitalization varied significantly, ranging from 2 to 25 cases per 100,000 person/years in studies that included individuals of all ages. The varying hospital admission criteria in different contexts may contribute to the discrepancy in the estimations. As individuals grew older, the rates of hospitalization significantly rose, with most cases observed in adults aged 50 years or older [4]. Hospitalization is more likely for older adults and individuals with weakened or suppressed immune systems. In our study, we found 5-fold increased odds of hospitalization among the immunosuppressed population. This increase might be related to risk stratification related to immunosuppression. The rise might also be related to clinicians maintaining a lower threshold for diagnosis and treatment in these groups. In Germany, the incidence rates of hospitalization varied from 31 per 100,000 individuals in people aged 60-64 years to 100 per 100,000 individuals in persons aged 80 years or beyond [4]. In our study, 355 individuals, accounting for 18.1% of the sample, needed to be admitted to the hospital because of HZ. The average age of hospitalized cases with HS HZ was greater than that of outpatients. Hospitalized cases exhibited a higher prevalence of underlying illnesses and immunosuppression. The logistic regression analysis revealed the risk factors that have an impact on hospitalization, including age, immunosuppression, hypertension, hematological disorders, transplantation, COPD, HZO, and disseminated disease. It is advisable to consider hospitalization for closer observation of individuals who have received allogenic stem cell transplants, particularly within the first 4 months after the transplant. Hospitalization is also recommended for hematopoietic stem cell transplant recipients who have moderate to severe graft versus host disease, transplant recipients undergoing aggressive antirejection therapy, individuals with suspected spread of infection to internal organs, and individuals with HZO or VZV retinitis [3]. In our study, HZO (5x increase) and disseminated involvement (15x increase) also increased hospitalization. Furthermore, it is advisable to carefully observe the development of diseases and the effectiveness of treatment in frail old adults [3]. In our study, the median duration of hospitalization was 8 days. Additional research indicated an average duration of 6.37–7.2 days for the whole stay [7]. On the other hand, an Italian study on hospitalized individuals with HZ found that their average stay lasted 23 days [15]. Interpreting these data proves challenging due to the difficulty in distinguishing between hospitalization caused by HS HZ or underlying factors such as immunosuppression, even though some instances necessitate extended hospital stays.

Individuals who are over 50 years old, have a moderate to severe rash or pain, have non-truncal involvement, and are immunocompromised should receive antiviral treatment for acute HZ. Commence antiviral treatment beyond 72 h following the appearance of a rash, provided there is observable evidence of new lesion development or the presence of motor, neurologic, or ocular problems. When compared directly, there is no discernible distinction between antiviral medications at treatment endpoints. When selecting an agent, one must consider elements such as the simplicity of dosage, the extent to which the body absorbs and utilizes it, and the financial implications, despite the fact that antiviral medications have comparable side effect profiles [3]. Patients admitted to the hospital predominantly favored acyclovir, while those receiving outpatient care primarily favored valacyclovir and brivudine, according to our study. The preferred antiviral treatment for uncomplicated HZ is the oral administration of acyclovir, valacyclovir, or famciclovir [7]. Besides antiviral and antibiotic treatment, doctors wrote about other types of treatment that mostly used topical drugs, such as drying agents, antivirals, antibiotics, steroids, and/or painkillers [7].

Out of the total number of patients in our study, 10 patients (0.51% of the sample) necessitated staying in the intensive care unit. We conducted a study over a period of 5 years and observed 7 occurrences of HZ, all of which resulted in death. During this entire study period, the HZ mortality rate was 0.36. All cases died within a hospitalization period of 5 to 63 days, and each case had a pre-existing disease. The mortality rate for HZ in a hospital setting varied from 0% to 36%. Mortality rates have consistently risen in correlation with advancing age. In the studies, the mortality rates linked to HZ varied between 0.017 and 0.465 per 100,000 person/years, with the majority of deaths occurring in people aged 60 or older [7]. The mortality rate due to HZ in the general population varies from 0.017 (Belgium, 1998–2007) to 0.465 (Sweden, 2006–2010) deaths per 100,000 person/years, with the majority of deaths occurring after the age of 60 [4]. The mortality rate due to HZ in Argentina varied between 0.0093 and 0.017. Among individuals above 15 years old, the mortality rate was 0–0.018 for males and 0.0047–0.027 for females. For patients over 80 years old, the mortality rate was greater, ranging from 0.105 to 0.448 [7]. The case fatality rate varied from 0% for HZO and uncomplicated HZ to 2.39% for diffused cases [7]. While the total number of deaths is very small, it is noteworthy that many deaths occur among older individuals. We cannot determine if this is the main cause of death or an intervening or contemporaneous cause.

Postherpetic neuralgia (PHN) is the most prevalent and debilitating consequence of HZ, affecting approximately 20% of individuals [16,17]. PHN can endure for a minimum of three weeks or months, and in some cases, it might remain for a year or more after the rash has healed [2]. The condition hampers the sleep, everyday functioning, work performance, and overall well-being of the person affected [17,18,19]. The prevalence of PHN following HZ varies from 5% to 30%, depending on factors such as study design, participant age distribution, and the specific definition used. Additionally, PHN affects around 50% of those over the age of 85 who have had HZ [3,4]. In immunocompetent patients, the incidence of PHN was 18%, while in immunocompromised patients, it was 22% [7]. Factors that increase the likelihood of developing PHN include prodromal symptoms, the intensity of pain and the expanse of the rash, advanced age, a weakened immune system, DM, and the presence of HZO [3]. In our study, 9.9% of the total population reported having PHN. The average age in the PHN group was substantially greater than that of HZ patients without PHN (63.5 ± 13.8 years vs. 54.0 ± 18.1 years). There is no discernible disparity in dermatome engagement between people afflicted with PHN and those without it. Logistic regression analysis identified age and the absence of previous antiviral treatments as risk factors for PHN. Currently, the effectiveness of antiviral medication in PHN is uncertain [20]. A small retrospective study involving 90 patients found that the majority of patients over the age of 65 who experienced PHN had not undergone antiviral treatment (62.1%) [21]. Patients with HZ who did not receive antiviral medications had a higher incidence of PHN, whereas those treated with acyclovir and brivudine had a lower incidence.

The incidence of HZ increased significantly across age groups. According to the Minnesota study in the United States, the annual increase corresponds to a 4.5-fold rise in the occurrence rate of HZ between 1945 and 2007 [5]. The implementation of varicella immunization, antiviral medication, or a shift in the occurrence of patients with weakened immune systems is unlikely to have contributed to the rise in cases [5]. Research demonstrated a consistent rise in the occurrence rates across all age categories prior to the implementation of the varicella vaccine program, and the upward trend persisted even after the introduction of the vaccination program. The temporal dynamics or onset of infectious diseases typically arise from societal, technological, viral, or environmental alterations, including climate change. The temporal increase was independent of age. An increase in the prevalence of risk factors, an increase in the use of immunosuppressive agents, or an increase in diagnosis due to improved access to healthcare and public awareness could partially explain it [3].

In this study, the incidence of PHN in Türkiye is 9.1%, and the hospitalization rate of HZ was 18.1%. When compared to neighboring countries, these figures offer helpful details about the regional burden of HZ and its complications. In Iran, a study conducted in Iran found hospitalization rates for HZ are reported to be around 10%, with higher rates in older populations and PHN incidence of approximately 15% among HZ patients [22]. In Greece, hospitalization rates range from 5% to 12%, depending on age and comorbidities, and PHN incidence ranges from 10% to 20%, increasing with age [23]. Differences between hospitalization rates between countries appear to be higher than those reported in neighboring countries, which may reflect differences in healthcare practices, accessibility, or population health status. Hospitalizations and the PHN management involve significant financial implications, both direct and indirect, highlighting the condition’s broad public health consequences. Direct healthcare expenditures correlate with prolonged hospital stays, specialized treatments including advanced pain management strategies, with costs significantly increasing among older adults and immunocompromised individuals [24,25]. The management of PHN often requires extended treatment durations, potentially lasting months or years, which consequently raises overall healthcare costs. PHN is linked to reduced productivity, lower quality of life, and a decline in autonomy. The aging global population is anticipated to lead to a rise in the prevalence and associated costs of HZ and PHN, thereby placing further strain on healthcare systems [25]. The main strategy to minimize the global health burden of VZV and HZ is through vaccination [25,26,27]. Vaccination, such as the recombinant zoster vaccine, serves as a cost-effective strategy to reduce the incidence of HZ and PHN thereby mitigating extended economic and public health issues. The National Immunization Program in Türkiye implemented a live varicella vaccine, administered as a single dose at 12 months of age, in February 2013. However, it is uncertain whether this vaccination has any impact on our research population [28]. No patients within our sample received either the varicella vaccine or the zoster vaccine. Vaccines for HZ are not accessible during the duration of this trial. Currently, the only zoster vaccine that has been licensed is available as a recombinant subunit vaccine [29]. A recombinant HZ vaccine is an effective and safe strategy for HZ prevention [30]. This vaccine has also been in private practice in Türkiye. Consequently, there is a lack of awareness about the vaccine among Turkish individuals. The HZ vaccine, with an affordable price, would be an option for older adults and high-risk groups.

The study we conducted has both limitations and merits. This study is a retrospective analysis. We have no chance to evaluate HZ patients who did not seek medical care. It is imperative to remove patients with incomplete medical data from our study’s methodology. We used ICD-10 diagnostic codes for HZ. All centers use the same coding practices; however, we underestimated some underlying conditions via this coding system or from medical records. We collected medical records of patients diagnosed with HZ from eight centers in seven different cities but did not represent the whole country. These centers are tertiary referral centers, and these centers represent severe cases, hospitalization, immunosuppression, and mortality. Recruiting from selective tertiary care centers introduces a selection bias that overrepresents severe cases. Nevertheless, this is the most extensive clinical case series from Türkiye, encompassing all pertinent information regarding HZ.

Recent decades have seen an increase in HZ occurrence in several countries, and projections indicate that the global population’s aging due to longer life expectancies will further amplify the impact of HZ on global health [6]. While HZ may not be given much importance in many countries, it is anticipated that the number of HZ patients will significantly rise in the coming decades due to the projected doubling of the population aged 60 years and older [4]. The significant and heightened load of HZ justifies the need to persistently observe HZ trends and associated problems. Healthcare practitioners and health regulators should prioritize the implementation of efficient follow-up, registration, treatment, and preventative measures against HZ [4]. Antiviral medication and hospitalization are effective healthcare measures that can be advantageous in fighting disease and treating complications of HZ, particularly in high-risk patients and older age groups [7]. Implementing vaccination programs for adults could effectively mitigate the occurrence of HZ disease and its associated sequelae within the most vulnerable populations. To gain a comprehensive understanding of the current situation, it would be beneficial to conduct more research that incorporates data from the post-vaccine period for pediatric varicella vaccine usage, as well as the pre- and post-COVID-19 pandemic. Moreover, additional research is required to investigate the economic impact of the condition, encompassing both direct and indirect expenses associated with the disease’s effect on quality of life.

## Figures and Tables

**Table 1:** Demographic characteristics, underlying diseases, dermatome involvement, treatment, and prognosis of patients with herpes zoster, categorized by the study year.

	2009 (n = 146)	2010 (n = 184)	2011 (n = 324)	2012 (n = 486)	2013 (n = 464)	2014 (n = 351)	TOTAL (n = 1955)
**Gender (female/male)**	73/73	91/93	157/167	238/248	242/222	209/142	1010/945
**Age (years, mean ± SD)** **Minimum–Maximum**	58.1 ± 18.1 19–93	55.3 ± 19.7 18–88	54.8 ± 17.7 18–92	56.0 ± 17.6 18–97	54.1 ± 18.0 18–88	53.4 ± 17.3 18–87	55.0 ± 17.9 18–97
**18–29 years**	14 (9.6%)	29 (15.7%)	40 (12.3%)	53 (10.9%)	69 (14.8%)	47 (13.4%)	252 (12.9%)
**30–39 years**	14 (9.6%)	20 (10.8%)	34 (10.5%)	42 (8.6%)	36 (7.7%)	36 (10.2%)	182 (9.3%)
**40–49 years**	11 (7.5%)	10 (5.4%)	33 (10.2%)	56 (11.5%)	57 (12.3%)	44 (12.5%)	211 (10.8%)
**50–59 years**	28 (19.2%)	29 (15.7%)	72 (22.2%)	101 (20.8%)	98 (21.1%)	69 (19.6%)	397 (20.3%)
**60–69 years**	36 (24.7%)	50 (27.1%)	74 (22.8%)	121 (24.9%)	107 (23.0%)	87 (24.8%)	475 (24.3%)
**70–79 years**	27 (18.5%)	28 (15.2%)	45 (13.8%)	78 (16.0%)	63 (13.6%)	52 (14.8%)	293 (15.0%)
**80–89 years**	14 (9.6%)	18 (9.8%)	23 (7.1%)	29 (5.9%)	34 (7.3%)	16 (4.5%)	134 (6.9%)
**>90 years**	2 (1.3%)	-	3 (0.9%)	6 (1.2%)	-	-	11 (0.6%)
**Immunosuppression**	29 (19.8%)	27 (14.6%)	67 (20.6%)	90 (18.5%)	80 (17.2%)	52 (14.8%)	345 (17.6%)
**Underlying condition**	58 (39.7%)	55 (29.9%)	132 (40.7%)	182 (37.4%)	164 (35.3%)	104 (29.6%)	695 (35.5%)
Hypertension	11 (7.5%)	17 (9.2%)	27 (8.3%)	42 (8.6%)	33 (7.1%)	16 (4.6%)	146 (7.5%)
Diabetes mellitus	4 (2.7%)	6 (3.2%)	25 (7.7%)	25 (5.1%)	22 (4.7%)	19 (5.4%)	101 (5.2%)
Hematological disorders	8 (5.5%)	15 (8.1%)	21 (6.5%)	33 (6.8%)	22 (4.7%)	21 (6.0%)	120 (6.1%)
Solid tumors	12 (8.2%)	4 (5.4%)	27 (8.3%)	25 (5.1%)	30 (6.5%)	20 (5.7%)	118 (6.0%)
Transplantation	1 (0.7%)	1 (0.5%)	10 (3.0%)	14 (2.9%)	10 (2.1%)	1 (0.3%)	37 (1.9%)
Coronary artery disease	6 (4.1%)	5 (2.7%)	9 (2.8%)	7 (1.4%)	19 (4.0%)	7 (2.0%)	53 (2.7%)
COPD	5 (3.4%)	6 (3.2%)	10 (3.0%)	10 (2.0%)	11 (2.4%)	4 (1.1%)	46 (2.3%)
Rheumatological	3 (2.0%)	4 (5.4%)	9 (2.8%)	15 (3.0%)	16 (3.4%)	11 (3.1%)	58 (2.9%)
Endocrinological	1 (0.7%)	2 (1.0%)	8 (2.4%)	4 (0.8%)	5 (1.0%)	3 (0.8%)	23 (1.2%)
Stroke	3 (2.0%)	-	1 (0.3%)	2 (0.4%)	4 (0.8%)	2 (0.6%)	12 (0.61%)
IBD	2 (1.3%)	1 (0.5%)	-	-	2 (0.4%)	1 (0.3%)	6 (0.3%)
Liver disorders	2 (1.3%)	1 (0.5%)	1 (0.3%)	5 (1.0%)	1 (0.2%)	-	10 (0.51%)
Renal disorders	3 (2.0%)	3 (1.6%)	4 (1.2%)	8 (1.6%)	5 (1.0%)	3 (0.8%)	26 (1.3%)
Neurological disorders	5 (3.4%)	1 (0.5%)	5 (1.5%)	9 (1.8%)	2 (0.4%)	-	22 (1.12%)
Pregnancy	-	-	1 (0.3%)	-	1 (0.2%)	2 (0.6%)	4 (0.2%)
**Dermatome involvement, n (%)**							
Cervical	35 (24.1%)	31 (16.8%)	58(17.9%)	96 (19.7%)	87 (18.7%)	72 (20.5%)	379 (19.4%)
Thoracal	49 (33.8%)	88 (47.8%)	171 (52.8%)	238 (48.9%)	243 (52.3%)	195 (55.5%)	984 (50.3%)
Lumbar	19 (13.1%)	24 (13.0%)	37 (11.4%)	68 (13.9%)	49 (10.5%)	31 (8.8%)	228 (11.7%)
Sacral	9 (6.2%)	8 (4.3%)	20 (6.2%)	22 (4.5%)	34 (7.3%)	16 (4.6%)	109 (5.6%)
Trigeminal	1 (0.7%)	4 (2.2%)	6 (1.8%)	18 (3.7%)	6 (1.3%)	7 (2.0%)	42 (2.1%)
HZO	16 (11.0%)	9 (4.9%%)	11 (3.4%)	16 (3.2%)	12 (2.6%)	9 (2.6%)	73 (3.7%)
Disseminated	2 (1.4%)	4 (2.2%)	4 (1.2%)	2 (0.4%)	4 (0.8%)	0	16 (0.8%)
Unknown	14 (9.6%)	17 (9.2%)	17 (5.2%)	26 (5.2%)	29 (6.3%)	21 (6.0%)	124 (6.3%)
**Postherpetic neuralgia, n (%)**	11 (7.5%)	31 (16.8%)	26 (8.0%)	53 (10.9%)	53 (11.4%)	21 (5.9%)	195 (9.9%)
**Hospitalization, n (%)**	42 (28.8%)	36 (19.6%)	78 (24.0%)	90 (18.5%)	71 (15.3%)	38 (10.8%)	355 (18.1%)
Length of hospital stay *	8 (6)	8 (5)	8 (6)	8 (9.75)	8 (5)	7 (7.25)	8 (6)
**ICU stay, n (%)**	0	0	2 (0.5%)	3 (0.6%)	3 (1.0%)	2 (1.5%)	-
**Antiviral use, n (%)**							
Acyclovir	45 (30.8%)	28 (15.2%)	69 (21.3%)	66 (13.6%)	67 (14.4%)	56 (15.9%)	331 (16.9%)
Valacyclovir	45 (30.8%)	71 (38.5%)	114 (35.1%)	224 (46.0%)	222 (47.8%)	160 (45.6%)	836 (42.7%)
Acyclovir + Valacyclovir	2 (1.3%)	5 (2.7%)	3 (0.9%)	5 (1.0%)	3 (0.6%)	2 (0.6%)	20 (1.0%)
Brivudine	21 (14.3%)	16 (8.7%)	17 (5.2%)	72 (14.8%)	87 (18.7%)	100 (28.4%)	313 (16.0%)
None	33 (22.6%)	64 (34.8%)	121 (37.3%)	119 (24.5%)	85 (18.3%)	33 (9.4%)	455 (23.2%)
**Antibiotic use, n (%)**							
Systemic antibiotic	13 (8.9%)	8 (4.3%)	22 (6.8%)	30 (6.2%)	15 (3.2%)	10 (2.8%)	98 (5.0%)
**Mortality**	0	0	3 (0.9%)	2 (0.4%)	1 (0.2%)	1 (0.3%)	7 (0.36%)

* Median (interquartile range).

**Table 2:** Underlying diseases, dermatome involvement, treatment, and prognosis of patients with herpes zoster, categorized by age groups.

	18–29 years (n = 252)	30–39 years (n = 182)	40–49 years (n = 211)	50–59 years (n = 397)	60–69 years (n = 475)	70–79 years (n = 293)	80–89 years (n = 134)	>90 years (n = 11)	TOTAL (n = 1955)
**Gender (female/male)**	132/120	94/88	125/86	215/182	225/250	146/147	64/70	9/2	1010/945
**Immunosuppression**	20 (7.93%)	19 (10.4%)	42 (19.9%)	89 (22.4%)	105 (22.2%)	54 (18.4%)	14 (10.4%)	2 (18.1%)	345 (17.6%)
**Underlying condition**	32 (12.7%)	32 (17.6%)	67 (31.7%)	143 (36.0)	214 (45.0%)	135(46.0%)	67 (50%)	5 (45.4%)	695 (35.5%)
Hypertension	-	1 (0.5%)	3 (1.4%)	19 (4.8%)	43 (9.0%)	44 (15.0%)	32 (23.9)	4 (36.3%)	146 (7.5%)
Diabetes mellitus	1 (0.4%)	-	9 (4.3%)	16 (4.0%)	38 (8.0%)	26 (8.8%)	10 (7.5%)	1 (9.0%)	101 (5.2%)
Hematological disorders	9 (3.6%)	8 (4.4%)	12 (5.7%)	32 (8.0%)	32 (6.7%)	19 (6.5%)	7 (5.2%)	1 (9.0%)	120 (6.1%)
Solid tumors	1 (0.4%)	4 (2.2%)	8 (3.8%)	24 (6.0%)	44 (9.3%)	30 (10.2%)	7 (5.2%)	-	118 (6.0%)
Transplantation	5 (2.0%)	3 (1.6%)	12 (5.7%)	9 (2.3%)	6 (1.3%)	2 (0.7%)	-	-	37 (1.9%)
Coronary artery disease	-	-	1 (0.5%)	3 (0.7%)	18 (3.8%)	16 (5.5%)	14 (10.4%)	1 (9.0%)	53 (2.7%)
COPD	1 (0.4%)	-	5 (2.4%)	4 (1.0%)	16 (3.4%)	13 (4.4%)	7 (5.2%)	-	46 (2.3%)
Rheumatological	4 (1.6%)	4 (2.2%)	5 (2.4%)	21 (5.3%)	17 (3.6%)	4 (1.4%)	2 (1.5%)	1 (9.0%)	58 (2.9%)
Endocrinological	3 (1.2%)	-	5 (2.4%)	4 (1.0%)	8 (1.7%)	2 (0.7%)	-	1 (9.0%)	23 (1.2%)
Stroke	1 (0.4)	2 (1.0%)	1 (0.5%)	1 (0.25%)	1 (0.2%)	2 (0.7%)	4 (2.9%)	-	12 (0.61%)
IBD	-	-	1 (0.5%)	3 (0.75%)	2 (0.4%)	-	-	-	6 (0.3%)
Liver disorders	-	1 (0.5%)	-	2 (0.5%)	2 (0.4%)	3 (1.0%)	2 (1.5%)	-	10 (0.51%)
Renal disorders	-	-	3 (1.4%)	3 (0.75%)	13 (2.7%)	6 (2.0%)	1 (0.7%)	-	26 (1.3%)
Neurological disorders	1 (0.4%)	-	-	5 (1.2%)	4 (0.8%)	4 (1.4%)	5 (3.7%)	1 (9.0%)	22 (1.12%)
Pregnancy	2 (0.8%)	2 (1.0%)	-	-	-	-	-	-	4 (0.2%)
**Dermatome involvement, n (%)**									
Cervical	49 (19.4)	36 (19.8%)	41 (19.4%)	70 (17.6%)	97 (20.4%)	53 (18.0%)	30 (22.4%)	3 (27.2%)	379 (19.4%)
Thoracal	114(45.2%)	84 (46.6%)	113(53.5%)	207(52.1%)	250(52.6%)	150(51.2%)	63 (47.0%)	3 (27.2%)	984 (50.3%)
Lumbar	32 (12.7%)	18 (9.9%)	17 (8.0%)	51 (12.8%)	50 (10.5%)	39 (13.3%)	19 (14.1%)	2 (18.2%)	228 (11.7%)
Sacral	15 (5.9%)	17 (9.3%)	14 (6.6%)	22 (5.5%)	18 (3.8%)	16 (5.5%)	7 (5.2%)	-	109 (5.6%)
Trigeminal	10 (3.9%)	4 (2.3%)	3 (1.4%)	8 (2.0%)	11 (2.3%)	3 (1.0%)	3 (2.3%)	-	42 (2.1%)
HZO	3 (1.1%)	5 (2.7%)	7 (3.3%)	19 (4.8%)	15 (3.2%)	18 (6.1%)	4 (3.0%)	2 (18.2%)	73 (3.7%)
Disseminated	2 (0.8%)	1 (0.5%)	2 (0.9%)	4 (1.0%)	4 (0.8%)	-	3 (2.2%)	-	16 (0.8%)
Unknown	27 (10.7%)	17 (9.3%)	14 (6.6%)	16 (4.0%)	30 (6.3%)	14 (4.8%)	5 (3.7%)	1 (9.0%)	124 (6.3%)
**Postherpetic neuralgia, n (%)**	7 (2.8%)	5 (2.7%)	7 (3.3%)	50 (12.6%)	58 (12.2%)	47 (16.0%)	21 (15.7%)	-	195 (9.9%)
**Hospitalization, n (%)**	27 (10.7%)	30 (16.5%)	25 (11.8%)	62 (15.6%)	98 (20.6%)	71 (24.2%)	39 (29.1%)	3 (27.2%)	355 (18.1%)
Length of hospital stay *	7 (4)	7 (2.75)	10 (6.25)	8 (5)	8.5 (7.75)	9 (6.5)	10 (8)	7.5 (15.25)	8 (6)
**ICU stay, n (%)**	-	-	-	2 (0.5%)	3 (0.6%)	3 (1.0%)	2 (1.5%)	-	10 (0.51%)
**Antiviral use, n (%)**									
Acyclovrir	40 (15.8%)	37 (20.3%)	28 (13.2%)	63 (15.9%)	76 (16.0%)	56 (19.1%)	26 (19.4%)	5 (45.4%)	331 (16.9%)
Valacyclovir	115 (45.6%)	68 (37.3%)	94 (44.5%)	187 (47.1%)	204 (42.9%)	115 (39.2%)	49 (36.5%)	4 (36.3%)	836 (42.7%)
Acyclovir + Valacycovir	1 (0.4%)	-	3 (1.4%)	5 (1.2%)	5 (1.0%)	5 (1.7%)	1 (0.7%)	-	20 (1.0%)
Brivudin	35 (13.9%)	27 (14.8%)	40 (18.9%)	76 (19.1%)	75 (15.8%)	43 (14.7%)	16 (11.9%)	1 (9.0%)	313 (16.0%)
None	61 (24.2%)	50 (27.5%)	46 (21.8%)	66 (16.6%)	115 (24.2%)	74 (25.2%)	42 (31.3%)	1 (9.0%)	455 (23.2%)
**Antibiotic use, n (%)**									
Systemic antibiotic	10 (3.9%)	1 (0.5%)	10 (4.7%)	14 (3.5%)	31 (6.5%)	16 (5.4%)	15 (11.2%)	1 (9.0%)	98 (5.0%)
**Mortality**	-	-	-	1 (0.25%)	3 (0.63%)	1 (0.34%)	2 (1.5%)	-	7 (0.36%)

* Median (interquartile range).

**Table 3:** Comparison between patients with or without immunosuppression based on age, gender, dermatome involvement, treatment, and prognosis.

	Immunosuppression (+) (n = 345)	Immunosuppression (−) (n = 1610)	*p*, OR; 95%CI
**Age (years, mean ± SD)**	57.9 ± 14.6	54.3 ± 18.5	*p* < 0.001
18–29 years	20 (5.8%)	232 (14.4%)	*p* < 0.001
30–39 years	19 (5.5%)	163 (10.1%)	*p* < 0.01
40–49 years	42 (12.2%)	169 (10.5%)	ns
50–59 years	89 (25.8%)	308 (19.1%)	*p* < 0.01
60–69 years	105 (30.4%)	370 (22.9%)	*p* < 0.01
70–79 years	54 (15.64%)	239 (14.9%)	ns
80–89 years	14 (4.0%)	120 (7.5%)	*p* < 0.05
>90 years	2 (0.6%)	9 (0.56%)	ns
**Gender (female/male)**	160/185	850/758	*p* < 0.05; OR 1.29; 95%CI: 1.02–1.64
**Dermatome involvement, n (%)**			
Cervical	57 (16.6%)	322 (20.0%)	ns
Thoracal	154 (44.8%)	827 (51.5%)	ns
Lumbar	48 (13.9%)	180 (11.2%)	ns
Sacral	23 (6.7%)	86 (5.4%)	ns
Trigeminal	11 (3.2%)	31 (1.9%)	ns
HZO	15 (4.3%)	58 (3.6%)	ns
Disseminated	12 (3.5%)	4 (0.25%)	*p* < 0.001; OR 14.5; 95%CI: 4.6–45.1
**Antiviral use, n (%)**			
Acyclovir	115 (33.3%)	215 (13.3%)	*p* < 0.001; OR 3.26; 95%CI: 2.50–4.26
Valacyclovir	158 (45.8%)	677 (42.1%)	ns
Brivudine	31 (9.0%)	282 (17.5%)	*p* < 0.01; OR 0.46; 95%CI: 0.31–0.68
None	34 (9.8%)	421 (26.2%)	*p* < 0.001; OR 0.3; 95%CI: 0.21–0.44
**Hospitalization, n (%)**	153 (44.3%)	203 (12.6%)	*p* < 0.001; OR 5.57; 95%CI: 4.26–7.28

**Table 4:** Comparison between hospitalized patients and outpatients based on age, gender, underlying disorders, dermatome involvement, treatment, and prognosis.

	Hospitalized (n = 355)	Ambulatory (n = 1600)	*p*, OR; 95%CI
**Age (years, mean ±SD)**	59.9 ± 17.4	53.9 ± 17.9	*p* < 0.001
**Gender (female/male)**	161/194	849/751	*p* < 0.01; OR 1.36; 95%CI: 1.07–1.72
**Immunosuppression**	153 (43.1%)	192 (12.0%)	*p* < 0.001; OR 5.57; 95%CI: 4.26–7.28
**Underlying condition**	272 (76.6%)	423 (26.4%)	*p* < 0.001; OR 9.10; 95%CI: 6.91–12.0
Hypertension	57 (16.0%)	89 (%5.6)	*p* < 0.001; OR 3.24; 95%CI: 2.23–4.69
Diabetes mellitus	33 (9.3%)	68 (4.2%)	*p* < 0.001; OR 2.30; 95%CI: 1.44–3.61
Hematological disorders	60 (16.9%)	60 (3.7%)	*p* < 0.01; OR 5.21; 95%CI: 3.50–7.76
Solid tumors	47 (13.2%)	71 (4.4%)	*p* < 0.001; OR 3.28; 95%CI: 2.17–4.92
Transplantation	21 (5.9%)	16 (1.0%)	*p* < 0.001; OR 6.21; 95%CI: 3.05–12.8
Coronary artery disease	18 (5.0%)	35 (2.2%)	*p* < 0.01; OR 2.38; 95%CI: 1.25–4.391
COPD	24 (6.7%)	22 (1.4%)	*p* < 0.001; OR 5.19; 95%CI: 2.75–9.84
Rheumatological	23 (6.5%)	35 (2.2%)	*p* < 0.001; OR 3.09; 95%CI: 1.72–5.49
Endocrinological	3 (0.8%)	20 (1.2)	ns
Stroke	4 (1.1%)	8 (0.5%)	ns
IBD	1 (0.3%)	5 (0.3%)	ns
Liver disorders	3 (0.8%)	7 (0.4%)	ns
Renal disorders	9 (2.5%)	17 (1.0%)	*p* < 0.05; OR 2.42; 95%CI: 1.07–5.47
Neurological disorders	11 (3.0%)	11 (0.7%)	*p* < 0.001; OR 4.61; 95%CI: 1.98–10.7
Pregnancy	1 (0.3%)	3 (0.2%)	ns
**Dermatome involvement, n (%)**			
Cervical	75 (21.1%)	304 (19.0%)	ns
Thoracal	138 (38.8%)	846 (52.8%)	ns
Lumbar	32 (9.0%)	196 (12.2%)	ns
Sacral	24 (6.7%)	85 (5.3%)	ns
Trigeminal	9 (2.5%)	33 (2.0%)	ns
HZO	34 (9.6%)	39 (2.4%)	*p* < 0.001; OR 423; 95%CI: 2.63–6.81
Disseminated	14 (3.9%)	2 (0.12%)	*p* < 0.001; OR 32.8; 95%CI: 7.4–145.0
**PHN**	31 (8.7%)	164 (10.2)	ns
**Antiviral use, n (%)**			
Acyclovir	186 (52.3%)	145 (9.0%)	*p* < 0.001; OR 11.0; 95%CI: 8.4–14.4
Valacyclovir	93 (26.2%)	743 (46.4%)	*p* < 0.001; OR 0.4; 95%CI: 0.31–0.52
Brivudine	7 (1.9%)	306 (18.5%)	*p* < 0.001; OR 0.08; 95%CI: 0.04–0.18
None	52 (14.6%%)	402 (25.2%)	*p* < 0.001; OR 0.51; 95%CI: 0.37–0.70
**Antibiotic use, n (%)**			
Systemic antibiotic	81 (22.8%)	1 (0.5%)	*p* < 0.001; OR 472; 95%CI: 65–3410

**Table 5:** Comparison between patients with or without PHN based on age, gender, underlying disorders, dermatome involvement, treatment, and prognosis.

	PHN (+) (n = 195)	PHN (−) (n = 1760)	*p*, OR; 95%CI
**Age (years, mean ±SD)**	63.5 ± 13.8	54.0 ± 18.1	*p* < 0.001
**Gender (female/male)**	110/85	900/860	ns
**Immunosuppression**	30 (15.4%)	315 (17.9%)	ns
**Underlying condition**	67 (34.3%)	628 (35.7%)	ns
Hypertension	14 (7.2%)	132 (%7.5)	ns
Diabetes mellitus	7 (3.6%)	94 (5.3%)	ns
Hematological disorders	10 (5.1%)	110 (6.2%)	ns
Solid tumors	9 (4.6%)	109 (6.2%)	ns
Transplantation	2 (1.0%)	35 (1.9%)	ns
Coronary artery disease	6 (3.0%)	47 (2.7%)	ns
COPD	3 (1.5%)	43 (2.4%)	ns
Rheumatological	7 (3.6%)	51 (2.9%)	ns
Endocrinological	5 (2.6%)	18 (1.0)	ns
Stroke	2 (1.0)	10 (0.5%)	ns
IBD	-	6 (0.3)	ns
Liver disorders	2 (1.0)	8 (0.4%)	ns
Renal disorders	2 (1.0)	24 (1.4%)	ns
Neurological disorders	2 (1.0)	20 (1.1%)	ns
Pregnancy	-	4 (0.2%)	ns
**Dermatome involvement, n (%)**			
Cervical	36 (18.5%)	343 (19.5%)	ns
Thoracal	107 (54.8%)	877 (49.8%)	ns
Lumbar	20 (10.2%)	208 (11.8%)	ns
Sacral	7 (3.6%)	102 (5.8%)	ns
Trigeminal	7 (3.6%)	35 (1.9%)	ns
HZO	8 (5.3%)	65 (3.7%)	ns
Disseminated	1 (0.5%)	15 (0.9%)	ns
**Antiviral use, n (%)**			
Acyclovir	21 (10.7%)	310 (17.6%)	*p* < 0.05; OR 0.56; 95%CI: 0.35–0.90
Valacyclovir	79 (40.5%)	757 (43.0%)	ns
Brivudine	15 (8.2%)	297 (16.8%)	*p* < 0.01; OR 0.41; 95%CI: 0.23–0.70
None	76 (38.9%)	379 (21.5%)	*p* < 0.001; OR 2.32; 95%CI: 1.70–3.17
**Hospitalization, n (%)**	31 (15.9%)	324 (18.4%)	ns

## Data Availability

The data presented in this study are available on request from the corresponding author.

## Abbreviations

HZ: Herpes Zoster, COPD: chronic obstructive pulmonary disorder; PHN: postherpetic neuralgia; VZV: Varicella-Zoster virus; HZO: Herpes Zoster Opthalmicus.
